# Blood as a route of transmission of uterine pathogens from the gut to the uterus in cows

**DOI:** 10.1186/s40168-017-0328-9

**Published:** 2017-08-25

**Authors:** Soo Jin Jeon, Federico Cunha, Achilles Vieira-Neto, Rodrigo C. Bicalho, Svetlana Lima, Marcela L. Bicalho, Klibs N. Galvão

**Affiliations:** 1 Department of Large Animal Clinical Sciences, College of Veterinary Medicine, University of Florida, Gainesville, FL 32610 USA; 2 Department of Animal Sciences, University of Florida, Gainesville, FL 32610 USA; 3 Department of Population Medicine and Diagnostic Sciences, College of Veterinary Medicine, Cornell University, Ithaca, NY 14850 USA; 4 Department of Clinical Sciences, Cornell University, Ithaca, NY 14850 USA; 5 D. H. Barron Reproductive and Perinatal Biology Research Program, University of Florida, Gainesville, FL 32610 USA

**Keywords:** Blood microbiota, Fecal microbiota, Uterine microbiota, Dairy cows, Droplet digital PCR, *Bacteroides heparinolyticus*, *Fusobacterium necrophorum*, Network analysis, Metagenomics, Uterine disease

## Abstract

**Background:**

Metritis is an inflammatory disease of the uterus caused by bacterial infection, particularly *Bacteroides*, *Porphyromonas*, and *Fusobacterium*. Bacteria from the environment, feces, or vagina are believed to be the only sources of uterine contamination. Blood seeps into the uterus after calving; therefore, we hypothesized that blood could also be a seeding source of uterine bacteria. Herein, we compared bacterial communities from blood, feces, and uterine samples from the same cows at 0 and 2 days postpartum using deep sequencing and qPCR. The vaginal microbiome 7 days before calving was also compared.

**Results:**

There was a unique structure of bacterial communities by sample type. Principal coordinate analysis revealed two distinct clusters for blood and feces, whereas vaginal and uterine bacterial communities were more scattered, indicating greater variability. Cluster analysis indicated that uterine bacterial communities were more similar to fecal bacterial communities than vaginal and blood bacterial communities. Nonetheless, there were core genera shared by all blood, feces, vaginal, and uterine samples. Major uterine pathogens such as *Bacteroides*, *Porphyromonas*, and *Fusobacterium* were part of the core genera in blood, feces, and vagina. Other uterine pathogens such as *Prevotella* and *Helcococcus* were not part of the core genera in vaginal samples. In addition, uterine pathogens showed a strong and significant interaction with each other in the network of blood microbiota, but not in feces or vagina. These microbial interactions in blood may be an important component of disease etiology. The copy number of total bacteria in blood and uterus was correlated; the same did not occur in other sites. *Bacteroides heparinolyticus* was more abundant in the uterus on day 0, and both *B. heparinolyticus* and *Fusobacterium necrophorum* were more abundant in the uterus than in the blood and feces on day 2. This indicates that *B. heparinolyticus* has a tropism for the uterus, whereas both pathogens thrive in the uterine environment early postpartum.

**Conclusions:**

Blood harbored a unique microbiome that contained the main uterine pathogens such as *Bacteroides*, *Porphyromonas*, and *Fusobacterium*. The presence of these pathogens in blood shortly after calving shows the feasibility of hematogenous spread of uterine pathogens in cows.

**Electronic supplementary material:**

The online version of this article (10.1186/s40168-017-0328-9) contains supplementary material, which is available to authorized users.

## Background

According to the World Health Organization, maternal sepsis from infection of the uterus (metritis) postpartum is still prevalent (~ 5%) in developing countries and accounted for 77,000 maternal deaths worldwide in 2000 [1]. The incidence of metritis in dairy cows is even greater (~ 20%) [2–4]; therefore, cows represent a good model for the study of uterine infections in large mammals. In our previous study, the uterine microbiota at calving was discriminated between cows that remained healthy and cows that later developed metritis, showing that the uterus begins to establish a bacterial community towards either health or disease shortly after calving [5]. Management strategies to control the source of bacterial contamination or to control the proliferation of pathogenic bacteria in the uterus such as vaccination [6] or the use of probiotics [7] could help prevent metritis in dairy cows.

It is widely believed that uterine bacteria ascend from the vagina or through the vagina from the environment or feces, when the cervix, which serves as an anatomical and immunological barrier, opens during parturition [8, 9]. Pathogens associated with the development of metritis such as *Bacteroides*, *Fusobacterium*, and *Porphyromonas* [5, 10, 11] are part of the normal flora of the rumen in cows [12] and are shed in feces; therefore, ascending uterine contamination from the environment could contribute to the development of metritis. The vaginal microbiota of beef and dairy cows have also been shown to harbor the main uterine pathogens such as *Bacteroides*, *Fusobacterium*, and *Porphyromonas* [13, 14]; therefore, ascending uterine contamination from the vagina is also possible. Nonetheless, one specific uterine pathogen, *Fusobacterium necrophorum* (*F. necrophorum*), is known to gain access to the circulation, probably during episodes of rumen acidosis, and cause liver abscesses in cows [15]. Interestingly, *F. necrophorum* is usually co-cultured with *Trueperella* (*Arcanobacterium*) *pyogenes* [15], another important uterine pathogen [8]. Furthermore, *Helcococcus ovis*, an emerging uterine pathogen [5, 11], was first reported to cause valvular endocarditis in cattle [16]. Therefore, hematogenous transmission must be considered as a possible route of dissemination of uterine pathogens in addition to ascending contamination from or through the vagina. Indeed, blood is a normal component of lochia; therefore, maternal blood is naturally transferred to the lumen of the uterus after birth. In cows, this mostly occurs because of degenerative vascular changes characterized by pyknosis followed by karyorrhexis of the endothelial cells and the cells of the tunica media of small blood vessels, changes that are observed within 24 h after calving [17]. Indeed, the endometrium becomes highly edematous during the first 24 h after calving [18], which is probably the result of the aforementioned vascular changes, hence leading to leakage of blood components into the uterine lumen. Although bacteria could be free-floating in blood, recent studies in mice and cows showed that bacteria could be transported to extraintestinal sites by mononuclear cells [19, 20]. Interestingly, translocation to extraintestinal sites was more common in mice in late gestation and shortly after parturition than in mice that were not pregnant or were in early or mid-lactation [19]. In cows as well as in other species, there is massive migration of leukocytes to the uterus with impending parturition and into the uterus after parturition [21, 22]; therefore, free-floating bacteria or bacteria engulfed by monocytes/macrophages could be readily transferred to the uterine lumen after calving.

The blood microbiome has not been investigated in peripartum dairy cows. Here, we hypothesized that cow’s blood would have a microbiome that would contain the main uterine pathogens such as *Fusobacterium*, *Bacteroides*, and *Porphyromonas*, therefore making the hematogenous route a feasible transmission route from the gut to the uterus. Given that microbiota in feces represents the distal portion of the gut microbiota [23], we sought to characterize bacterial communities in the blood, feces, and uterine samples to examine how these communities are related to each other. We collected samples from the same 12 individuals within 60 min after calving to minimize exogenous bacterial contamination and at 2 days after calving to examine the change in bacterial community. Blood was collected aseptically from the jugular vein. Because the vaginal microbiome also harbors uterine pathogens, we included the vaginal microbiome from a previous study [14] in our analysis for comparison. This study provides insight into the origin of uterine bacteria as well as the potential role of the gut and blood microbiota in uterine disease.

## Results

### Characteristics of the study samples

Blood, fecal, and uterine samples were collected from the same 12 Holstein dairy cows on day 0 (the day of calving) to minimize exogenous bacterial contamination and on day 2 (2 days postpartum) to examine the change in bacterial community. The V4 region of the bacterial 16S ribosomal RNA (rRNA) gene was PCR-amplified from all 72 samples, and sequencing was performed on the Illumina MiSeq platform. Samples that failed quality control were excluded for taxonomic classification; therefore, 19 blood samples (9 on day 0 and 10 on day 2), 22 fecal samples (10 on day 0 and 12 on day 2), and 20 uterine samples (10 on day 0 and 10 on day 2) were analyzed. 16S rRNA sequencing resulted in 6,818,977 reads, with an average of 85,651 ± 5888 reads (standard error of the mean) from blood samples, 156,566 ± 62,878 reads (standard error of the mean) from fecal samples, and 87,358 ± 4956 reads (standard error of the mean) from uterine samples. Rarefaction curves of 61 samples at the minimum cutoff of 97% sequence identity nearly reached a plateau, which indicates that sampling depth is sufficient to characterize bacterial communities (Additional file 1: Figure S1).

For comparison, vaginal data were obtained from a previously published study [14], which were generated using the same sequencing technique and quality control as other samples. In our analysis, we used data from vaginal samples collected on day − 7 (7 days prepartum) from 105 Holstein dairy cows that were different from the cows used for collection of samples from the uterus, feces, and blood. Although samples collected from different individuals have been used to compare the microbiome from different body sites such as the oral cavity, the gut, and the human placenta [24], these samples do not allow for direct comparisons between body sites such as comparison of means or evaluation of correlations between individual taxon. Although vaginal samples from day 0 were also available, those would be contaminated with uterine discharge, therefore not being able to differentiate between the uterine and vaginal microbiome.

### Dissimilarity of blood, fecal, and uterine microbiota

**Fig. 1:**
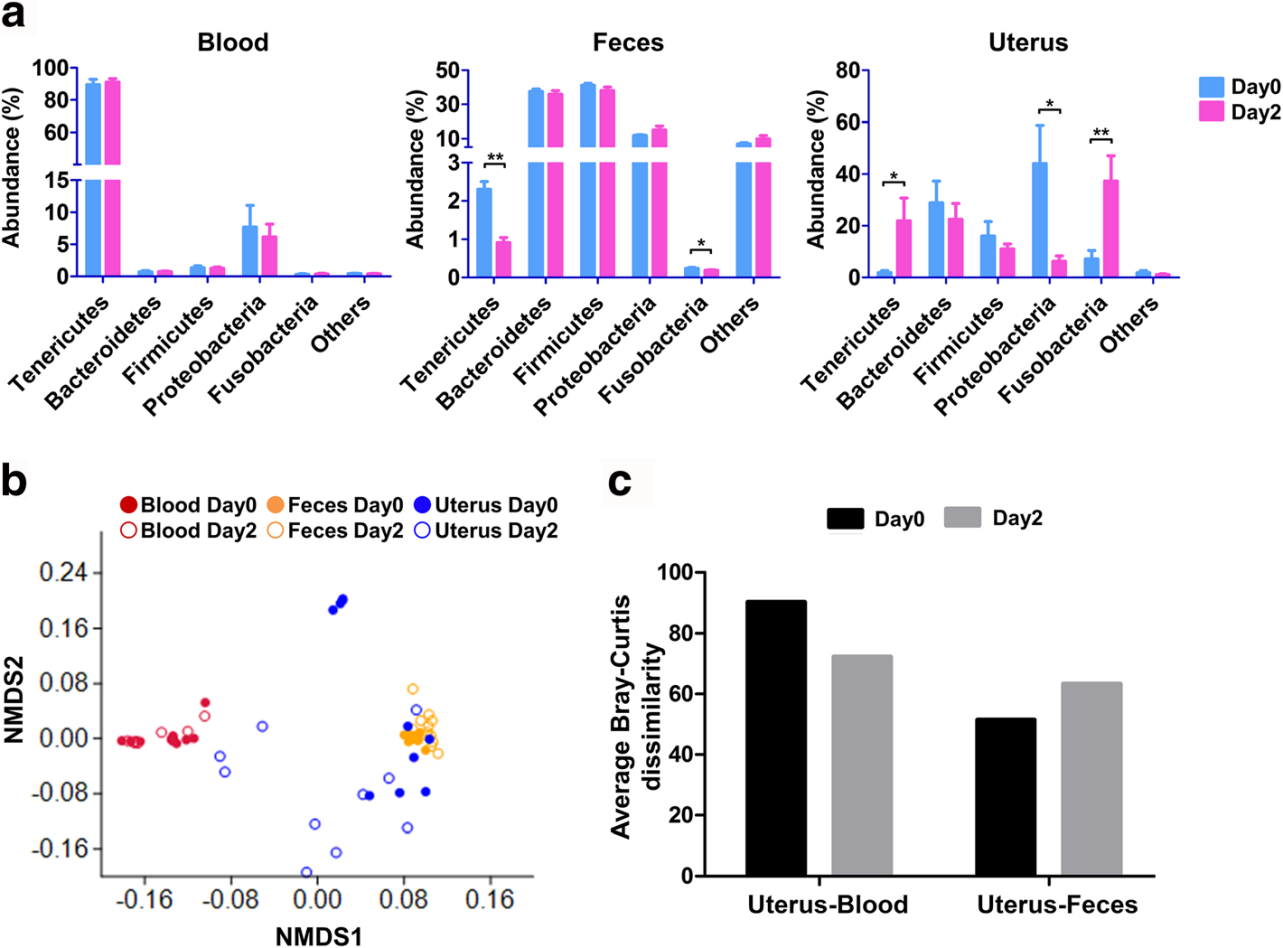
Characteristics of blood, fecal, and uterine microbiota. **a** Relative abundance of the five major phyla in the blood, feces, and uterus. The bars represent the means and standard error of the mean, and the asterisks indicate the statistical significance between day 0 and day 2 (Wilcoxon test, *P ≤ 0.05 and **P < 0.01). **b** NMDS plot of Bray-Curtis dissimilarity between samples from the blood (red), feces (orange), and uterus (blue) on day 0 (filled circles) and day 2 (empty circles). Bray-Curtis dissimilarity was calculated using the phylum level abundance of bacteria. Groups by body habitats (blood, feces, and uterus) were significantly different by a PERMANOVA analysis on Bray-Curtis dissimilarity (*P* = 0.0001; stress = 0.13). **c** The overall average Bray-Curtis dissimilarity between body habitats by SIMPER analysis at the phylum level (see Additional file 3: Table S1 for details)

To identify bacterial community structure and the change in bacterial abundance, we examined the relative abundance of bacterial phyla in the blood, feces, and uterine samples on day 0 and day 2 (Fig. 1a). Tenericutes, Bacteroidetes, Firmicutes, Proteobacteria, and Fusobacteria were the five most abundant phyla in all samples, which accounted for 99.6% in blood microbiota, 91.4% in fecal microbiota, and 98.5% in uterine microbiota. In blood, microbiota appeared to be stable from day 0 to day 2, with a high abundance of Tenericutes (mean relative abundance of 90.3%), which contributed to low diversity as shown in the Chao1 and Shannon indices (Additional file 2: Figure S2); nonetheless, numerous bacteria belonging to Proteobacteria (mean relative abundance of 6.9%), Firmicutes (mean relative abundance of 1.3%), and 24 others (< 1%) were also detected in the blood. In feces, Firmicutes and Bacteroidetes were the most abundant phyla, followed by Proteobacteria. These major phyla remained stable from day 0 to day 2, while rare phyla such as Tenericutes (2.3 vs. 0.9%, *P* < 0.01) and Fusobacteria (0.24 vs. 0.18%, *P* = 0.02) significantly decreased. On the other hand, uterine microbiota was highly dynamic from day 0 to day 2 by increasing Tenericutes (1.9 vs. 21.9%, *P* = 0.02) and Fusobacteria (7.2 vs. 37.3%, *P* < 0.01) and decreasing Proteobacteria (44.1% vs. 6.2%, *P* = 0.04). However, there were no significant differences in the species richness and diversity between day 0 and day 2 (Additional file 2: Figure S2) because of individual variations in bacterial abundance. To visualize the community similarity at the phylum level, the non-metric multi-dimensional scaling (NMDS) ordination was performed on the Bray-Curtis dissimilarity (Fig. 1b). The NMDS showed distinct clusters for blood and feces: the bacterial community profiles of blood grouped to the left of the NMDS plot and the bacterial community profiles of feces grouped to the right of the NMDS. The bacterial community profiles of the uterus were relatively divergent from each other and localized between the bacterial community profiles of blood and feces, with the community being closer to feces on day 0 and shifting towards blood on day 2. These bacterial community profiles from blood, feces, and uterus were significantly different by sample types according to PERMANOVA analysis of the Bray-Curtis dissimilarity (*P* = 0.0001). To confirm the result in the NMDS, the community dissimilarity was also evaluated in the similarity percentage (SIMPER) analysis of Bray-Curtis dissimilarity (Fig. 1c). SIMPER analysis of blood and fecal microbiota compared with uterine microbiota demonstrated that uterine microbiota was more similar to fecal microbiota (index of 51.4 on day 0 and 63.4 on day 2) than blood microbiota (index of 90.4 on day 0 and 72.3 on day 2). This high dissimilarity between the uterus and blood was attributed to the dominance of Tenericutes in the blood (Additional file 3: Table S1D and F). In addition, SIMPER analysis within each group, between day 0 and day 2, showed the overall average Bray-Curtis dissimilarity of 68.6 for uterine microbiota, 7.9 for blood microbiota, and 13.9 for fecal microbiota, suggesting that uterine microbiota is greatly dynamic during this period, while blood and fecal microbiota are relatively stable. Taxa contributing to the dissimilarity between groups and within groups are listed in Additional file 3: Table S1A–G.

### Similarity of bacterial community composition in blood, feces, and uterine samples

**Fig. 2:**
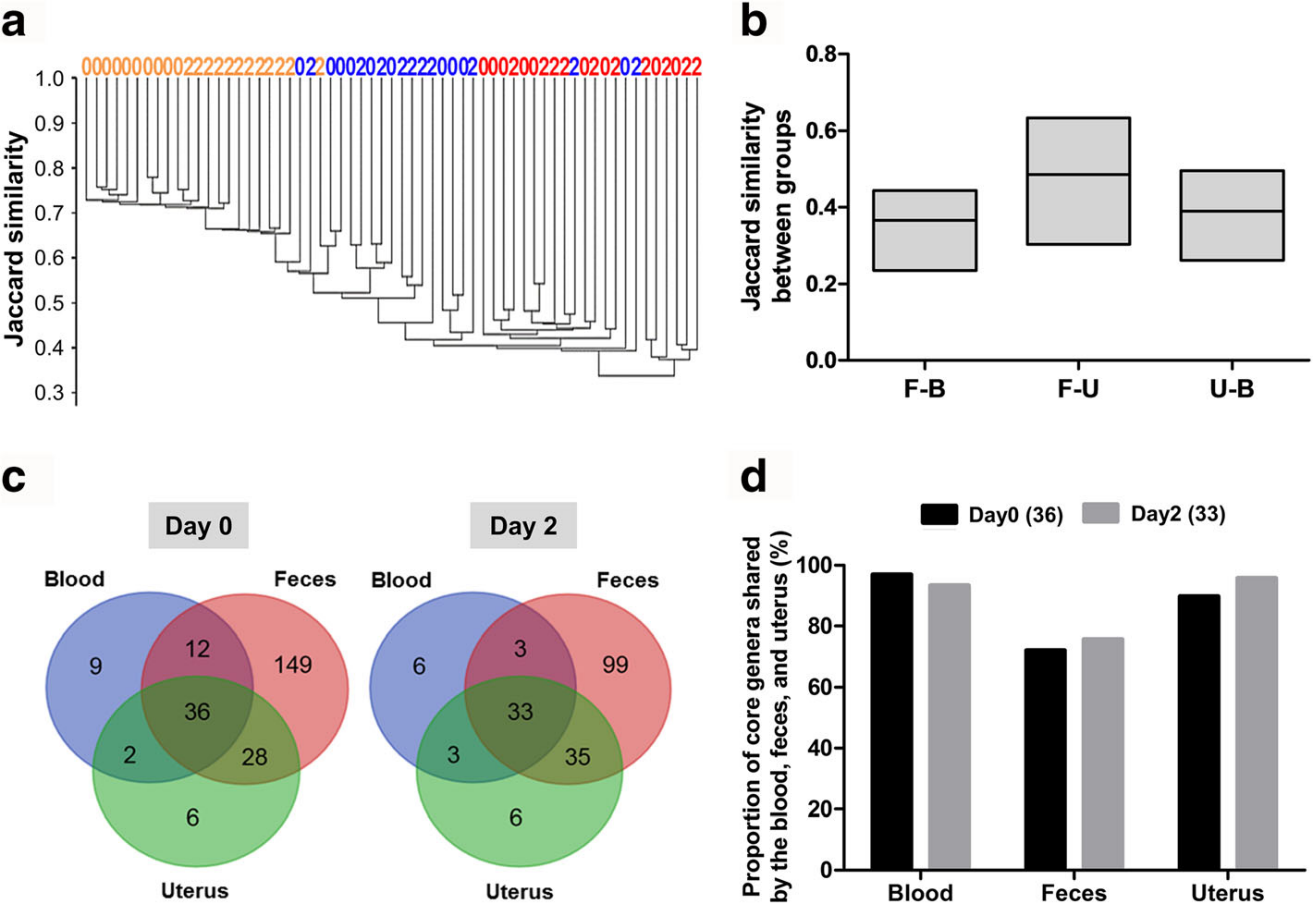
Similarity in bacterial community composition. **a** UPGMA cluster based on the Jaccard similarity at the genus level (correlation coefficient 0.95). **b** Jaccard similarity between body habitats: F-B feces-blood, F-U feces-uterus, and U-B uterus-blood. The floating bars show the minimum and maximum values, and the horizontal line inside the bars indicates the mean value. **c** Venn diagrams showing the number of core genera in blood, feces, and uterine samples on day 0 and day 2. Core genera are defined as genera that are found in all dairy cows. **d** Total relative abundance represented by core genera shared by blood, feces, and uterus. The figure shows the total relative abundance represented by the core genera found in every body site on day 0 (*n* = 36) and day 2 (*n* = 33) in relation to the bacterial community found in each body site. That is, the 36 core genera found in every body site represent a total relative abundance of 97.0% of the bacterial community found in blood on day 0

To measure the similarity of bacterial community composition between microbiota from the blood, feces, and uterus, bacterial profiles at the genus level were analyzed using the Jaccard similarity and unweighted pair-group method with an arithmetic average (UPGMA). Consistent with the NMDS analysis, the UPGMA clustering analysis revealed a significant difference by body habitats (*P* = 0.0001), where uterine samples appeared to be more similar to feces, compared to blood (Fig. 2a), with three exceptions. One uterine sample on day 0 and two uterine samples on day 2 were more similar to blood samples. Interestingly, fecal samples on day 0 tended to separate from fecal samples on day 2 (*P* = 0.067), indicating some shift in bacterial composition in the gut after calving. This shift seems to result in the decline of diversity of fecal microbiota as shown in the Shannon index (Additional file 2: Figure S2). Next, we calculated the Jaccard similarity between groups (Fig. 2b), which also shows the high degree of similarity between fecal and uterine microbiota. On the other hand, it should be noted that the Jaccard similarity index within the group was relatively lower in the blood and uterine microbiota when compared to fecal microbiota (Fig. 2a). This illustrates individual variation in blood and uterine microbiota composition. For this reason, we focused on core genera that were found in all individuals. The core genera and their relative abundance for each group are listed in Additional file 4: Table S2A–D. Using Venn diagrams, we found 36 core genera on day 0 and 33 core genera on day 2 overlapping by the blood, feces, and uterine samples (Fig. 2c). Core genera accounted for 97.0% of all blood bacteria on day 0 and 93.3% on day 2, 72.2% of all fecal bacteria on day 0 and 75.8% on day 2, and 89.8% of all uterine bacteria on day 0 and 95.8% on day 2 (Fig. 2d; Additional file 4: Table S2E and F). This suggests that these genera can persist across body habitats, becoming key components of bacterial communities of blood, feces, and uterus.

### Association of uterine pathogens with blood and fecal microbiota

**Fig. 3:**
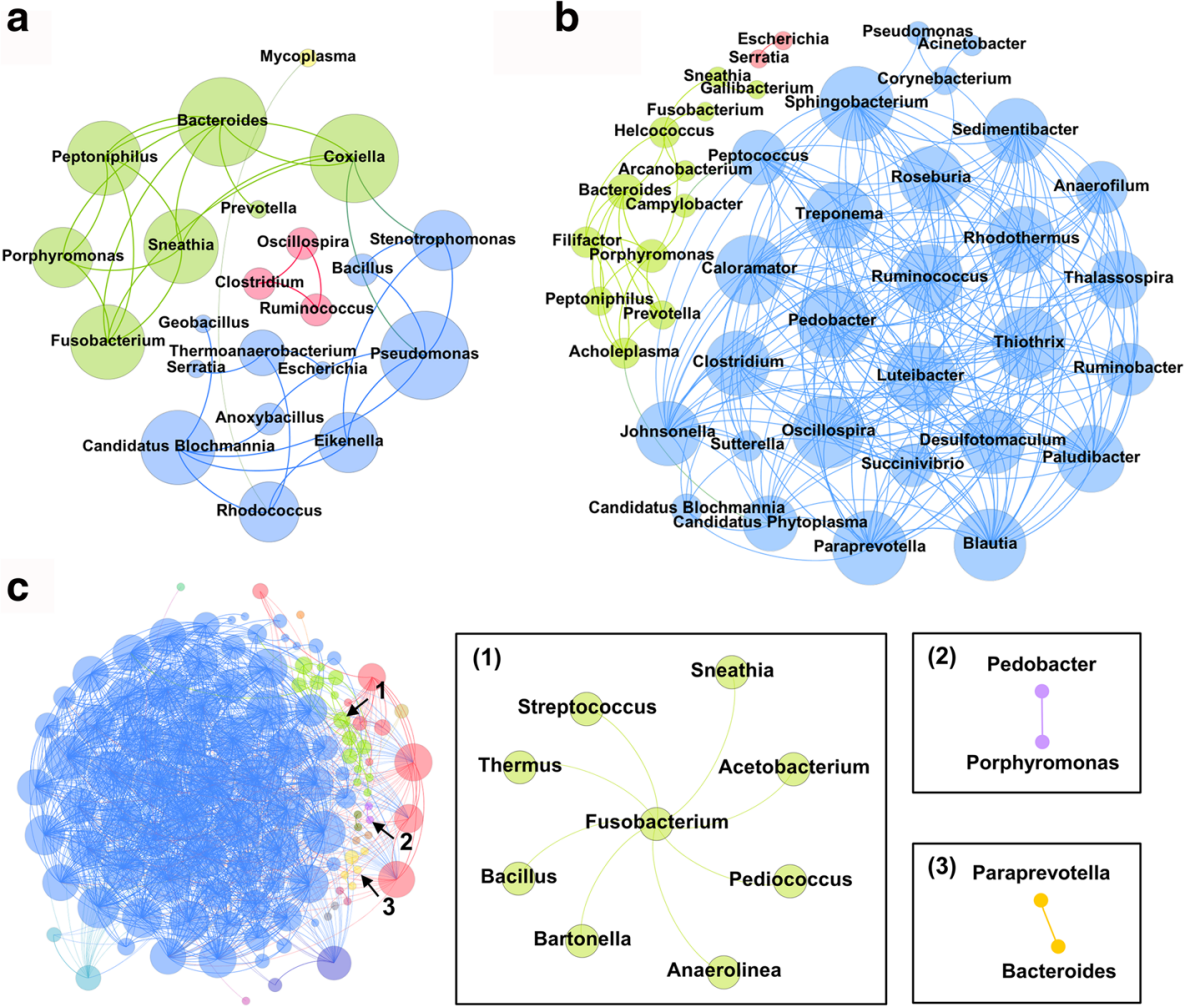
The network of co-occurring core genera within the body habitat: **a** blood, **b** uterus, and **c** feces. The nodes represent the core genera, and the size of each node is proportional to the degree (the number of connections). The edges stand for strong (Spearman’s correlation coefficient *r*_s_ < − 0.7 or *r*_s_ > 0.7) and significant (*P* < 0.01) correlations between core genera. The blood, uterus, and feces revealed 4, 4, and 14 modularity structures in the network, respectively. The nodes and edges are colored based on modularity structure: 1st modularity structure (blue), 2nd modularity structure (green), 3rd modularity structure (red), 4th modularity structure (yellow), and so on. The boxes (**c**) show the magnified views for co-occurrence patterns of pathogenic bacteria involved in uterine disease: (1) *Fusobacterium*, (2) *Porphyromonas*, and (3) *Bacteroides*

To explore bacterial interactions within body habitats, we used network analysis based on strong (Spearman’s *r*_s_ < − 0.7 or *r*_s_ > 0.7) and significant (*P* < 0.01) correlations of core genera found both on day 0 and day 2 (Fig. 3; see Additional file 5: Table S3A–C for details). In this network, it is assumed that co-occurring core genera interact with each other, either positively or negatively. The blood network consisted of 22 nodes (core genera) and 36 edges (relations) with an average degree (the mean number of connections per node) of 3.3 (Fig. 3a). The average clustering coefficient (the degree to which nodes tend to cluster together) was 0.68 and the modularity (the degree to which nodes have dense connections to each other within a sub-community, but have sparse connections in other sub-communities) was 0.53. According to the modularity algorithm [25], blood core genera were partitioned into four modularity structures (i.e., sub-communities), where major uterine pathogens such as *Bacteroides*, *Fusobacterium*, and *Porphyromonas* [5] were part of the same sub-community and had positive correlations. In addition, *Prevotella* and *Peptoniphilus*, which were part of the same sub-community, are also believed to contribute to the development of metritis [5]. *Helcococcus*, an emerging uterine pathogen [5, 11], was not part of the same sub-community, although it was a core bacterium in the blood (Additional file 5: Table S3A). On the other hand, *Mycoplasma* which was the most abundant genus in the blood (average 90.5%) lacked a bacterial interaction and only showed a negative correlation with *Rhodococcus*. The network of uterine bacterial community revealed 42 nodes and 267 edges with an average degree of 12.71 (Fig. 3b). The average clustering coefficient was 0.79, and the modularity was 0.19, forming four modularity structures in the network. Similar to the blood network, major uterine pathogens such as *Bacteroides*, *Fusobacterium*, and *Porphyromonas* along with other uterine pathogens such as *Prevotella*, *Helcococcus*, *Filifactor*, *Peptoniphilus*, *Campylobacter*, and *Arcanobacterium* [5, 11] were found to belong to the same sub-community (green nodes) and had positive correlations (Additional file 5: Table S3B). Interestingly, *Coxiella*, which is not believed to be a uterine pathogen but has high prevalence in the uterus on day 0, and *Sneathia*, which is associated with uterine health [5], were also part of the same sub-community (green nodes). This finding indicates a strong biological interaction that may be associated with survival in blood and transport to the uterus. Meanwhile, a majority of non-pathogenic bacteria were found in another sub-community (blue nodes). The network of fecal bacterial community showed 116 nodes, 1330 edges, and an average degree of 22.93 (Fig. 3c). The average clustering coefficient was 0.72 and the modularity was 0.19, with 14 modularity structures in the fecal network. Unlike the blood and uterine network, *Bacteroides*, *Fusobacterium*, and *Porphyromonas* were not part of the same sub-community, indicating no biological interactions. For example, *Bacteroides* was correlated with *Paraprevotella*, *Porphyromonas* was correlated with *Pedobacter*, and *Fusobacterium* was correlated with many bacteria including *Sneathia*, *Anaerolinea*, *Bartonella*, *Thermus*, *Acetobacterium*, *Pediococcus*, *Bacillus*, and *Streptococcus*. Taken together, clinically important bacteria associated with uterine disease showed similar correlation patterns in blood and uterus, whereas the same did not occur in feces. Nonetheless, all major and minor uterine pathogens were found in feces both on day 0 and on day 2 (Additional file 5: Table S3C).

### Comparison of blood, fecal, and uterine microbiota with vaginal microbiota

**Fig. 4:**
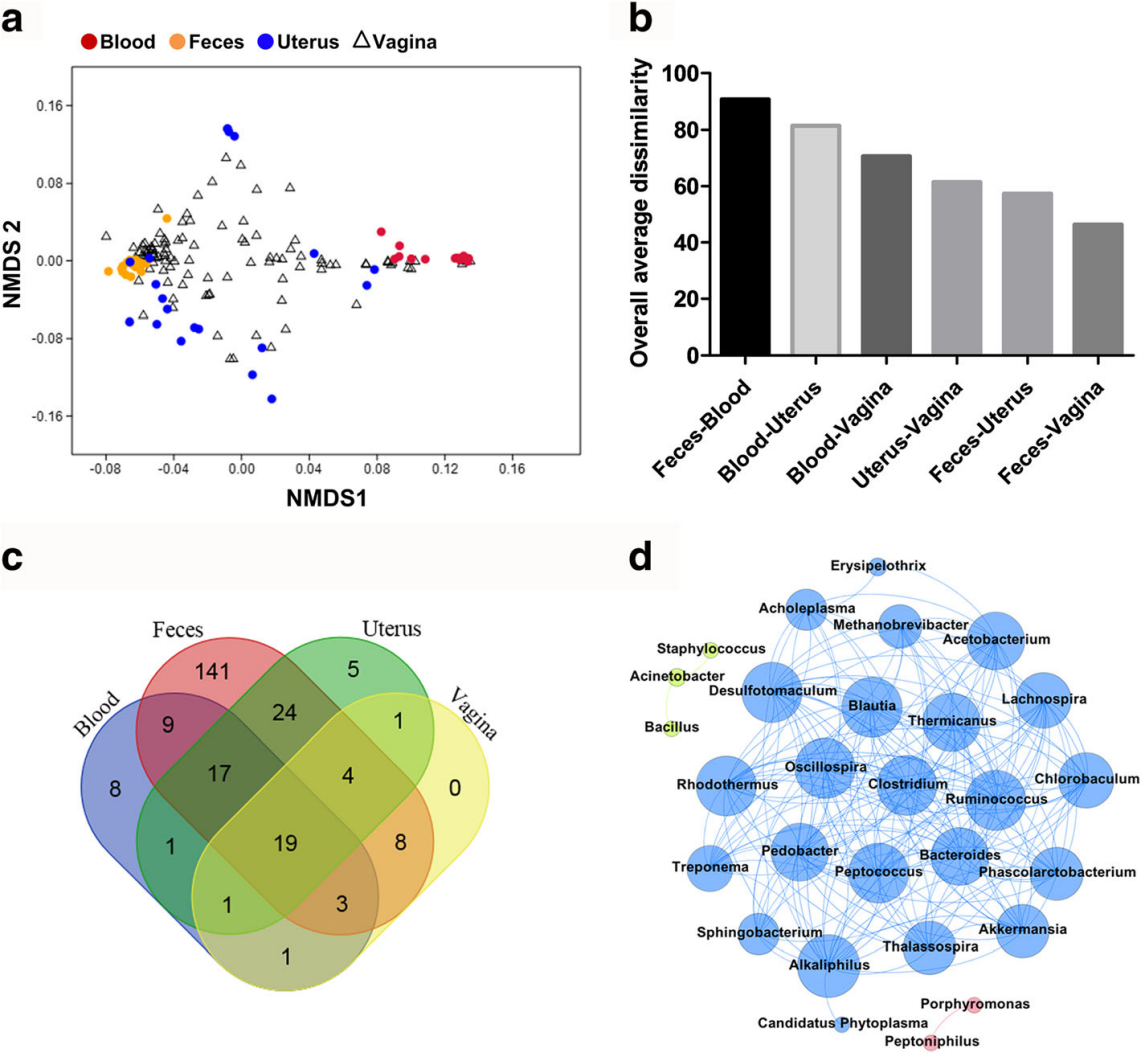
Comparison of blood, fecal, and uterine microbiota with vaginal microbiota. **a** NMDS plot of Bray-Curtis dissimilarity at the phylum level (PERMANOVA *P* = 0.0001, permutation = 9999, stress = 0.16). **b** The overall average Bray-Curtis dissimilarity between body habitats by SIMPER analysis at the phylum level. **c** Venn diagram showing the number of core genera. **d** Network analysis of vaginal microbiota. The nodes represent the vaginal core genera at day − 7. The edges indicate strong (Spearman’s *r*_s_ < − 0.7 or *r*_s_ > 0.7) and significant (*P* < 0.01) correlations between core genera. The nodes and edges are colored based on modularity structure

The NMDS plot of Bray-Curtis dissimilarity at the phylum level presented significant differences in bacterial communities from the blood, feces, uterus, and vagina (Fig. 4a; PERMANOVA *P* = 0.0001). Similar to the bacterial community profiles of the uterus, the vaginal bacterial communities were spread between the clusters of bacterial community profiles of blood and feces. Despite high variability, a large number of vaginal samples were closely clustered with feces in the NMDS plot. In addition, according to the SIMPER analysis, vaginal bacterial communities showed the least dissimilarity with fecal bacterial communities when compared to those from the blood and uterus (Fig. 4b). Interestingly, uterine bacterial communities were more similar to fecal bacterial communities than to vaginal bacterial communities. As shown in a Venn diagram illustrating the number of core genera of four body habitats (Fig. 4c), the vaginal core genera were mostly detected in the feces, but these core genera were also present in the blood and uterus. Although the main uterine pathogens such as *Bacteroides*, *Porphyromonas*, and *Fusobacterium* were found in all vaginal samples, other pathogens such as *Prevotella*, *Helcococcus*, *Filifactor*, *Campylobacter*, and *Arcanobacterium* were not (Additional file 4: Table S2D). The network of the vaginal bacterial community, based on strong (Spearman’s *r*_s_ < − 0.7 or *r*_s_ > 0.7) and significant (*P* < 0.01) correlations of core genera that were found on day − 7, were comprised of 28 nodes and 192 edges, with an average degree of 13.71 and the average clustering coefficient of 0.87 (Fig. 4d). Unlike the networks seen in the blood and uterus, no interaction was observed in the vagina among the uterine pathogens *Bacteroides*, *Porphyromonas*, and *Fusobacterium*. Overall, our analyses show high similarity between bacterial communities from the vagina and feces.

### Absolute quantification of bacteria

**Fig. 5:**
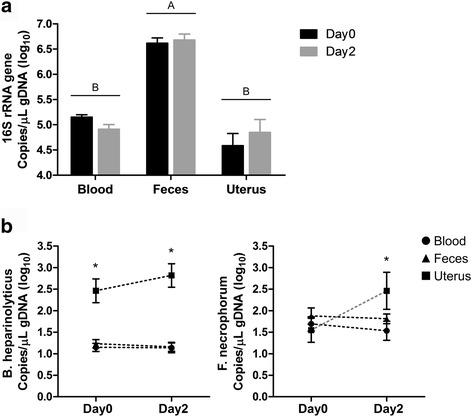
Absolute quantification of bacteria using ddPCR. **a** The total number of bacteria from the blood, feces, and uterus. Different letters (A, B) indicate a statistical significance among groups (one-way ANOVA, *P* ≤ 0.05). **b** The number of *Bacteroides heparinolyticus* and *Fusobacterium necrophorum*. The asterisks indicate a statistical significance among groups on each day 0 and day 2 (one-way ANOVA, *P* ≤ 0.05). All data represent means and standard error of the mean. Symbols: blood (circle), feces (triangle), and uterus (square)

To measure the total amount of bacteria in each habitat, we quantified 16S rRNA gene copies in genomic DNA (gDNA) samples from the blood, feces, and uterus of dairy cows on day 0 and day 2 using droplet digital PCR (ddPCR) (Fig. 5a). The mean 16S rRNA gene copies in blood were 5.15 ± 0.05 (log) on day 0 and 4.91 ± 0.09 (log) on day 2, with a tendency to decline from day 0 to day 2 (Wilcoxon test, *P* = 0.06). The mean 16S rRNA gene copies in feces were 6.62 ± 0.10 (log) on day 0 and 6.68 ± 0.12 (log) on day 2, with no significant difference between day 0 and day 2. The mean 16S rRNA gene copies in uterine samples were 4.59 ± 0.24 (log) on day 0 and 4.85 ± 0.25 (log) on day 2, with no significant difference from day 0 to day 2. In a comparison among the blood, feces, and uterus, the mean 16S rRNA gene copy in feces was significantly higher (*P* < 0.01) than those in the blood and in the uterus, and there was no significant difference between the blood and uterus; however, the mean 16S rRNA gene copy in the uterus tended to be lower (*P* = 0.07) than in the blood. This result demonstrates that despite the establishment of a bacterial community, the abundance of uterine microbiota was not fully developed by day 2.

To assess if the microbiota from one body site affected the microbiota from another site, we evaluated the correlations between the 16S rRNA gene copies in blood and uterus and between feces and uterus in cows that had samples from all three sites. We observed a tendency for a negative correlation (Spearman’s *r*_s_ = −0.69, *P* = 0.06) in the 16S rRNA gene copies between the uterus and blood (Additional file 6: Figure S3), meaning that blood bacteria decreased as uterine bacteria increased. This result also supports the idea of bacterial transport from the blood to the uterus. The correlations between feces and uterus (Spearman’s *r*_s_ = 0.60, *P* = 0.12) and between feces and blood (Spearman’s r_s_ = 0.05, *P* = 0.91) were not significant. The lack of correlation is probably because of the immensely greater bacterial biomass in the gut compared with other body sites; therefore, transfer of bacteria from the gut to the uterus may be more related to the integrity of physical barriers such as the vulva, vagina, and cervix, and transfer of bacteria from the gut to blood may be more related to the integrity of the gastrointestinal epithelium.

Previous metagenomic studies identified *Bacteroides heparinolyticus* and *Fusobacterium necrophorum* as uterine pathogens because these bacteria were more prevalent in the uterus of dairy cows with metritis, compared with those in that of the healthy cows [5, 26]. Therefore, to determine if uterine pathogens are present in blood and feces and, if they are, how many of them occupy their habitats, we used ddPCR for copy numbers of *B. heparinolyticus* and *F. necrophorum* (Fig. 5b). The mean copy numbers of *B. heparinolyticus* in uterine samples were 2.46 ± 0.28 (log) on day 0 and 2.82 ± 0.27 (log) on day 2, which were significantly greater (*P* < 0.01) than those in the blood and feces, with mean copy numbers of 1.15 ± 0.10 (log) on day 0 and 1.14 ± 0.11 (log) on day 2 for blood and mean copy numbers of 1.23 ± 0.10 (log) on day 0 and 1.16 ± 0.11 (log) on day 2 for feces. With regard to *F. necrophorum*, there was no statistical difference (*P* > 0.30) on day 0 among sample types, where the mean copy number was 1.69 ± 0.17 (log) for blood, 1.88 ± 0.19 (log) for feces, and 1.55 ± 0.28 (log) for uterine samples. Meanwhile, on day 2, the abundance of *F. necrophorum* was elevated in the uterus at 2.46 ± 0.43 (log), which was significantly more abundant (*P* = 0.03) than in the blood at 1.53 ± 0.22 (log) and tended to be higher (*P* < 0.10) than in the feces at 1.81 ± 0.11 (log). Both *B. heparinolyticus* and *F. necrophorum* showed an increasing trend in the uterus, although not significant. On the other hand, *B. heparinolyticus* and *F. necrophorum* remained at low abundance in blood and feces. Taken together, uterine pathogens were detected in blood and feces, but the uterine environment in early postpartum seems to provide the ideal conditions for them to thrive.

## Discussion

The current paradigm of the origin of uterine bacteria is that physical barriers are compromised during parturition, which allows for bacteria to ascend the genital tract from the vagina or through the vagina from the environment as well as the animal’s skin and feces [8, 9]. Nonetheless, as stated before, uterine pathogens such as *F. necrophorum* and *Trueperella pyogenes* [27] cause liver abscess and *Helcococcus ovis* causes valvular endocarditis [16]; therefore, a hematogenous route of colonization of the uterus cannot be discounted. Previously, we reported that the uterus had an established microbiome within 60 min of calving [5], which indicates that colonization occurred before or shortly after calving either by ascending contamination from or through the vagina, via the bloodstream, or both. Herein, we showed that blood harbored a unique microbiome that contained the main uterine pathogens such as *Bacteroides*, *Fusobacterium*, and *Porphyromonas*. The presence of uterine pathogens in feces and blood indicate the feasibility of a hematogenous spread of bacteria from the gut to the uterus. Nevertheless, ascending contamination of the uterus cannot be discarded because the vagina also harbors the main uterine pathogens. Interestingly, other uterine pathogens such as *Prevotella*, *Helcococcus*, *Filifactor*, *Campylobacter*, and *Arcanobacterium* were not part of the core vaginal microbiome. In addition, vaginal microbiota was distinct from uterine microbiota on NMDS plot (Fig. 4a), although both microbiota showed a similarity with fecal microbiota. Thus, these results indicate that both vaginal and uterine microbiota are influenced by the gut microbiota, as suggested in a previous study [28]. Nonetheless, it is worth noting that vaginal samples were collected from a different group of cows, which limits our ability to perform direct comparisons between the uterine and vaginal microbiomes. Therefore, sampling within the same animals is required in future studies to confirm our findings.

Previous studies have shown that network analysis is a powerful tool to investigate microbial interactions in complex environments such as soil and water [29, 30]. Thus, we applied network analysis to our samples to find bacterial genera that are important in the structure of their microbiota. Interestingly, uterine pathogens such as *Bacteroides*, *Porphyromonas*, and *Fusobacterium* formed similar networks in blood and uterus, despite the difference in microbiota abundance and composition (Additional file 7: Figure S4). The same was not observed in the vagina or feces. It is not clear at this point why this network is formed in blood, but it may be an important factor for their transmission to other body sites such as the uterus or liver. Of particular interest was the fact that *Coxiella* was found to be part of the blood network that included uterine pathogens. *Coxiella* is a bacterium that infects and multiplies inside of monocytes and has a tropism for the uterus [5, 31]; therefore, it is likely that the influx of leukocytes to the uterine lumen after calving contributes to the high prevalence of *Coxiella* on day 0. The correlation of *Coxiella* with uterine pathogens indicates that uterine pathogens may also be transported and transferred into the uterine lumen by monocytes. Although gut microbes were observed in blood leukocytes in cows in mid-lactation, uterine pathogens were not found in all cows [20]; therefore, presence of uterine pathogens in blood leukocytes shortly after calving should be investigated. Indeed, a study showed that bacterial translocation to extraintestinal sites was more common in late gestation and shortly after parturition than in the non-pregnant state or in early or mid-lactation [19]. Nonetheless, free-floating bacteria in blood could also be transferred to the uterus because of the degenerative vascular changes that occur shortly after calving [17].

Herein, we used ddPCR to quantify uterine-specific pathogens and all bacteria. Droplet digital PCR is considered a third-generation PCR technology and has been shown to be more accurate than the real-time PCR [32, 33]. We found that the total number of bacteria was lower in the uterus than in blood and feces on day 0 (Fig. 5a), in spite of high species richness and diversity (Additional file 2: Figure S2). This shows that blood is a reasonably abundant source of bacteria for seeding of other tissues. The observation of higher abundance of *B. heparinolyticus* in the uterus than in blood or feces at calving indicates a tropism of this bacterium for the uterus. It is not clear how this bacterium concentrates in the uterus but the same was not observed for *F. necrophorum*, although *F. necrophrum* also showed an adaptation for the uterine environment early postpartum. The negative correlation in total bacteria between the uterus and blood (Additional file 6: Figure S3) supports the idea of bacterial transport from the blood to the uterus via blood leukocytes, but the synergism among uterine pathogens in blood and their specific mechanisms of invasion of the uterus warrants further investigation.

## Conclusions

The blood, feces, vagina, and uterus have unique environments, and thereby, unique structures of bacterial communities were observed depending on body sites. Nonetheless, ordination and cluster analysis revealed that fecal bacterial communities are closely related to vaginal and uterine bacterial communities. Additionally, high abundance of core genera was shared by blood, fecal, vaginal, and uterine samples. More importantly, major uterine pathogens such as *Bacteroides*, *Porphyromonas*, and *Fusobacterium* and other uterine pathogens such as *Prevotella* and *Helcococcus* were part of the core genera in blood samples. Interestingly, although major uterine pathogens such as *Bacteroides*, *Porphyromonas*, and *Fusobacterium* were also part of the core genera in vaginal samples, other uterine pathogens such as *Prevotella* and *Helcococcus* were not part of the core genera in vaginal samples, which indicates that the blood may be the most important route of transmission of some uterine pathogens. Furthermore, uterine pathogens formed similar networks in blood and uterus, which may be an important factor for transmission, and warrants further investigation. The copy number of total bacteria in blood was correlated with the total bacteria in the uterus. On the other hand, the copy number of total bacteria in feces and uterus and feces and blood were not correlated. The copy number of total bacteria was higher in feces than in blood and uterus. In contrast, *B. heparinolyticus* was more abundant in the uterus on day 0, and both *B. heparinolyticus* and *F. necrophorum* were more abundant in the uterus than in the blood and in the feces on day 2. Our findings indicate that bacteria originating from the gut may be translocated to the uterus via the bloodstream. This study shows the feasibility of hematogenous spread of uterine pathogens in cows, although it does not exclude the possibility of direct fecal contamination or contamination from the vagina. In fact, direct fecal contamination or contamination from the vagina are likely to occur as well.

## Methods

### Animals and sampling

Holstein cows (*n* = 12) from a commercial dairy in Central Florida milking 5000 cows were used in this study. Blood, feces, and uterine samples were collected from the same individuals quickly after calving (within 60 min of calving; mean time = 20 min; SD = 14 min) to avoid or minimize the chance for contamination of the uterus from the environment ascending through the vagina. As part of the routine management, cows were changed from a high-fiber to a high-concentrate diet after calving, which leads to changes in the rumen and fecal microbiome [34], which could lead to changes in the blood microbiome. Therefore, we collected samples 2 days after calving to capture shifts in each separate microbiome. Cows were followed until 8 days postpartum for the diagnosis of metritis, characterized by fetid red-brownish watery uterine discharge, as previously reported [5]. Because there are no major differences in the uterine microbiome between healthy and metritic cows at calving and at 2 days postpartum [5] and because we wanted to focus on the source of the uterine contamination rather than the differences between metritic and healthy cows, we included six cows that later developed metritis and six cows that remained healthy in the study. All the cows were healthy at the time of sampling because the clinical signs of metritis did not develop until 6 ± 2 days postpartum [5].

Uterine samples were collected using a sterile swab (Har-VetTM McCullough Double-Guarded Uterine Culture Swab) as previously described [5]. Blood samples were collected from the jugular vein using vacutainer tubes with EDTA after surgically prepping a 150-cm^2^ area over the vein with iodine scrub and alcohol-soaked gauze pads. Fecal samples were collected from the rectum using sterile cotton-tipped swabs. Samples were stored at − 80 °C until DNA extraction.

Because this study aimed to confirm our hypothesis that gut bacteria could be transported to the uterus via the bloodstream, we did not sample the vagina. Nonetheless, for completeness, we compared our metagenomic data with vaginal data from a previous study as the reference [14]. Vaginal samples were collected on day − 7 (7 days prepartum) from 105 Holstein dairy cows that were different from the cows used for collection of other samples, using sterile cotton-tipped swabs.

### DNA extraction

The gDNA was isolated from uterine swabs using the QIAamp DNA Mini kit (Qiagen), from 400 μl of blood using the QIAamp DNA Blood Mini kit (Qiagen), and from 200 mg of feces using the QIAamp DNA Stool Mini kit (Qiagen). The steps were performed as directed by the manufacturer with a modification; all samples were incubated with 400 μg of lysozyme for 1 h at 37 °C to maximize bacterial DNA extraction. The DNA concentrations of samples were measured using NanoDrop^®^ ND-2000 (NanoDrop Technologies). The gDNA from vaginal swabs was extracted using the PowerSoil DNA Isolation Kit (MO BIO Laboratory Inc., Carlsbad, CA) after disruption of the sample using a bead beater homogenizer (Mini-Beadbeater-8, Biospec Products).

### Metagenomic sequencing

All the samples were sequenced, by our collaborators from Cornell University, using the same technique. The V4 hypervariable region of the 16S rRNA gene was amplified by PCR as previously described [35]. PCR products were tagged with a 12 bp error-correcting Golay barcodes. The 5′ barcoded amplicons were prepared in triplicate using 10 μM of primer 515F and 806R, 1× GoTaq Green Master Mix (Promega), 1 mM MgCl_2_, and DNA template as follows: an initial denaturing step at 94 °C for 3 min, followed by 35 cycles of 94 °C for 45 s, 50 °C for 1 min, and 72 °C for 90 s, and a final elongation step at 72 °C for 10 min. Replicate amplicons were pooled and purified with a QIAquick PCR Purification Kit (Qiagen), followed by electrophoresis to visualize PCR products. Purified amplicon was quantified using the Quant-iT™ PicoGreen® dsDNA Assay Kit (Life Technologies Corporation) to normalize the concentration of all DNA libraries. Normalized libraries were pooled and sequenced using the MiSeq reagent kit V2-300 cycles on the MiSeq platform (Illumina Inc.). The reads were demultiplexed and filtered in each sample, allowing a single mismatch in index recognition, a quality score of 30, and a minimum length of 100 nt. Taxonomy was assigned using the Metagenomics workflow based on an Illumina-curated version of the Greengenes database.

### Metagenomic and statistical analysis

Sequencing depth was evaluated using rarefaction curves in the Metagenomics RAST (https://metagenomics.anl.gov/) with the following parameters: annotation source Greengenes, maximum e-value cutoff 1e^− 5^, minimum identity % cutoff 97%, and minimum alignment length cutoff 100 bp. The Chao1 and Shannon indices were calculated in the R “fossil” and “vegan” packages, respectively (http://www.r-project.org). The relative abundance of bacterial phyla was compared between day 0 and day 2 using the Wilcoxon test. To represent the distance between samples, the NMDS of Bray-Curtis dissimilarity was carried out using PAST3 (http://folk.uio.no/ohammer/past/), in which non-parametric multivariate analysis of variance (PERMANOVA) was used to test significant difference among groups. To measure a difference in bacterial communities between groups and to identify which taxa are primarily responsible for the difference, the SIMPER analysis was conducted in the PAST3. To compare microbiota composition, the UPGMA clustering analysis was performed at the genus level based on Jaccard similarity using PAST3. The Jaccard similarity index was calculated in pairwise comparisons of the communities. Venn diagrams showing the number of core genera in blood, fecal, and uterine samples were created using the Bioinformatics & Evolutionary Genomics (http://bioinformatics.psb.ugent.be/webtools/Venn/). To understand the interrelationships of core genera within body habitats, co-occurrence patterns of core genera were evaluated in the network interface using pairwise Spearman’s rank correlations based on bacterial abundance. Strong (Spearman’s *r*_s_ < − 0.7 or *r*_s_ > 0.7) and significant (*P* < 0.01) correlations between core genera were considered a valid co-occurrence event. In the network, the nodes represent core genera and edges indicate relations among nodes. The topology of the network including average node connectivity, clustering coefficient, and modularity was calculated [36] and was visualized in the ForceAtlas2 algorithm using the Gephi (http://gephi.org) [37, 38].

### Droplet digital PCR

Absolute quantification of bacteria was examined by ddPCR using a DNA binding dye (EvaGreen) according to the manufacturer’s instructions. Universal 16S primers which were designed by Clifford et al. [39] were used for the identification of all bacteria, and species-specific primers for *B. heparinolyticus* and *F. necrophorum* were designed in this study using Primer3 (Additional file 8: Table S4). The ddPCR reaction mixture contained 10 μl Supermix (Bio-Rad), 250 nM primers, and gDNA (~ 40 ng) in a final volume of 20 μl and combined with 20 μl of droplet generation oil (Bio-Rad), which partitioned into approximately 20,000 droplets in the QX200 droplet generator (Bio-Rad). The droplets generated from each sample were amplified by PCR on the PTC-100 (Bio-Rad) with the following condition: 95 °C for 10 min, 40 cycles of 94 °C for 30 s, 55 °C for 45 s, and 72 °C for 50 s, followed by 72 °C for 5 min and a hold at 4 °C. All samples were run in duplicate. After amplification, each droplet was read by the QX200 droplet reader (Bio-Rad) to count the number of positive and negative droplets, and target DNA molecules were presented as copies per microliter by the QuantaSoft™ software (Bio-Rad). The original concentration of the target DNA was log_10_-transformed in copies per 1 μL of gDNA and was analyzed by ANOVA using JMP Pro 13. For statistical analysis, differences with *P* ≤ 0.05 were considered significant, and differences with 0.05 < *P* ≤ 0.10 were considered to have a tendency towards statistical significance.

## Additional files

Additional file 1: Figure S1. Rarefaction curves of 61 samples from blood, feces, and uterine swabs. Analysis was performed in the Metagenomics RAST with the following parameters: annotation source Greengenes, maximum e-value cutoff 1e^− 5^, minimum identity % cutoff 97%, and minimum alignment length cutoff 100 bp. (TIFF 3996 kb)

Additional file 2: Figure S2. Alpha diversity of blood, fecal, and uterine bacterial communities. (A) Chao1 and (B) Shannon index. A box shows the 25th and 75th percentiles and the horizontal line inside the box indicates the median. The whiskers of the box indicate the largest and smallest values. Different letters indicate statistical significance among the blood, feces, and uterus (ANOVA, *P* ≤ 0.05). The asterisks indicate statistical significance between 0 and 2 days postpartum within the group (Wilcoxon test, *P* < 0.01). (TIFF 1623 kb)

Additional file 3: Table S1. Similarity of percentages analysis of Bray–Curtis dissimilarity within and between body habitats. (A) Blood on day 0 and blood on day 2, (B) feces on day 0 and feces on day 2, (C) uterus on day 0 and uterus on day 2, (D) uterus on day 0 and blood on day 0, (E) uterus on day 0 and feces on day 0, (F) uterus on day 2 and blood on day 2, and (G) uterus on day 2 and feces on day 2. (XLSX 27 kb)

Additional file 4: Table S2. Core bacterial genera and its abundance at each body habitat (A) blood, (B) feces, (C) uterus, and (D) vagina. There were 36 core genera on day 0 (E) and 33 core genera on day 2 (F) that were present in all blood, feces, and uterine samples. (XLSX 79 kb)

Additional file 5: Table S3. Core genera (found together on day 0 and day 2) and their correlations in (A) blood, (B) uterus, and (C) feces. Nodes used in the network are highlighted in yellow. (XLSX 81 kb)

Additional file 6: Figure S3. Association between blood and uterine microbiota. (A) Total bacteria in the blood and uterus (Spearman’s *r*_s_ = 0.69, *P* = 0.06). (B) Total bacteria in the feces and uterus (Spearman’s *r*_s_ = 0.60, *P* = 0.12). (C) Total bacteria in the feces and blood (Spearman’s *r*_s_ = 0.05, *P* = 0.91). Blood, feces, and uterine samples were collected from the same individual cows on day 0, and total bacteria were measured using universal primers in ddPCR. (TIFF 1927 kb)

Additional file 7: Figure S4. Genus-level microbiota abundance. (A) Blood bacterial genera with a relative abundance > 0.1%. (B) Fecal bacterial genera with a relative abundance > 1%. (C) Uterine bacterial genera with a relative abundance > 1%. The asterisks indicate statistical significance between 0 and 2 days postpartum (Wilcoxon test, *P < 0.05, **P < 0.01). The arrows represent increases (blue) and decreases (red) in abundance. (PPTX 147 kb)

Additional file 8: Table S4. Primers used in droplet digital PCR. (PDF 145 kb)

Additional file 9: Table S5. Metadata. (XLSX 17 kb)
