# Supplementary figures and images for: Blood as a route of transmission of uterine pathogens from the gut to the uterus in cows
Source: Microbiome. 2017 Aug 25;5:109. doi: 10.1186/s40168-017-0328-9 (PMC5574159; doi:10.1186/s40168-017-0328-9)

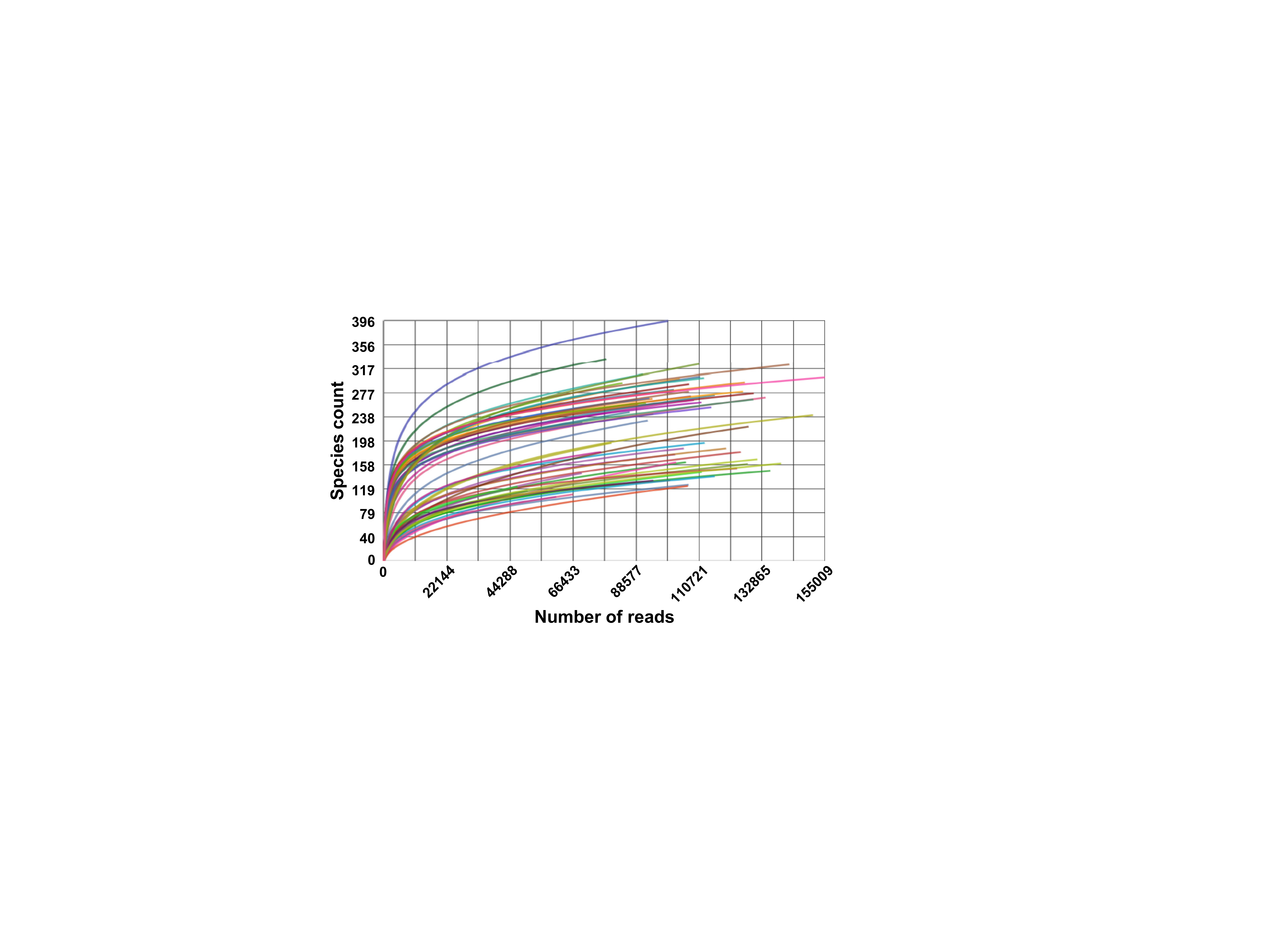

Supplement: Supplementary file 1 — Rarefaction curves of 61 samples from blood, feces, and uterine swabs. Analysis was performed in the Metagenomics RAST with the following parameters: annotation source Greengenes, maximum e-value cutoff 1e− 5, minimum identity % cutoff 97%, and minimum alignment length cutoff 100 bp. (TIFF 3996 kb) [file 40168_2017_328_MOESM1_ESM.tif]

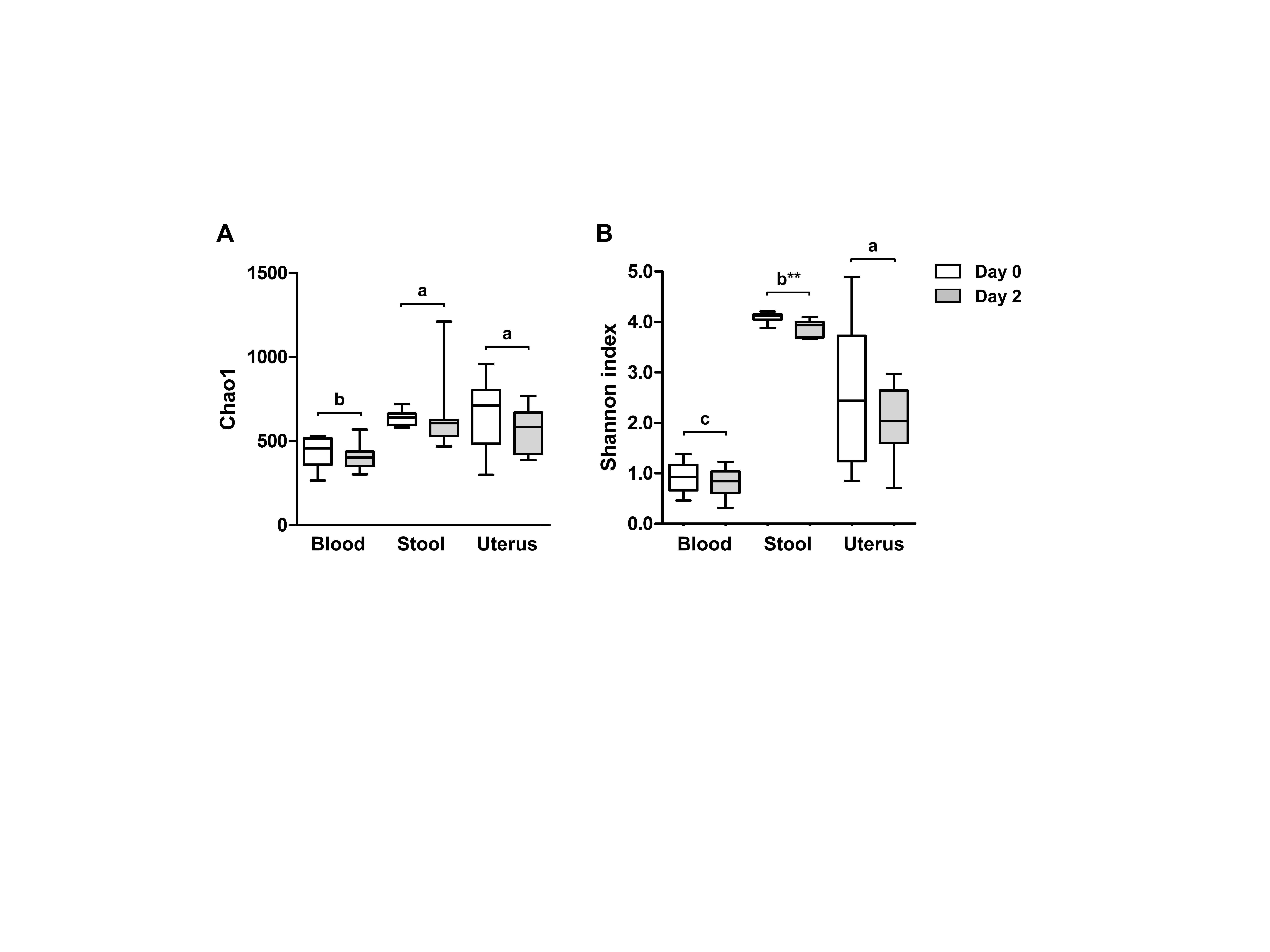

Supplement: Supplementary file 2 — Alpha diversity of blood, fecal, and uterine bacterial communities. (A) Chao1 and (B) Shannon index. A box shows the 25th and 75th percentiles and the horizontal line inside the box indicates the median. The whiskers of the box indicate the largest and smallest values. Different letters indicate statistical significance among the blood, feces, and uterus (ANOVA, P ≤ 0.05). The asterisks indicate statistical significance between 0 and 2 days postpartum within the group (Wilcoxon test, P < 0.01). (TIFF 1623 kb) [file 40168_2017_328_MOESM2_ESM.tif]

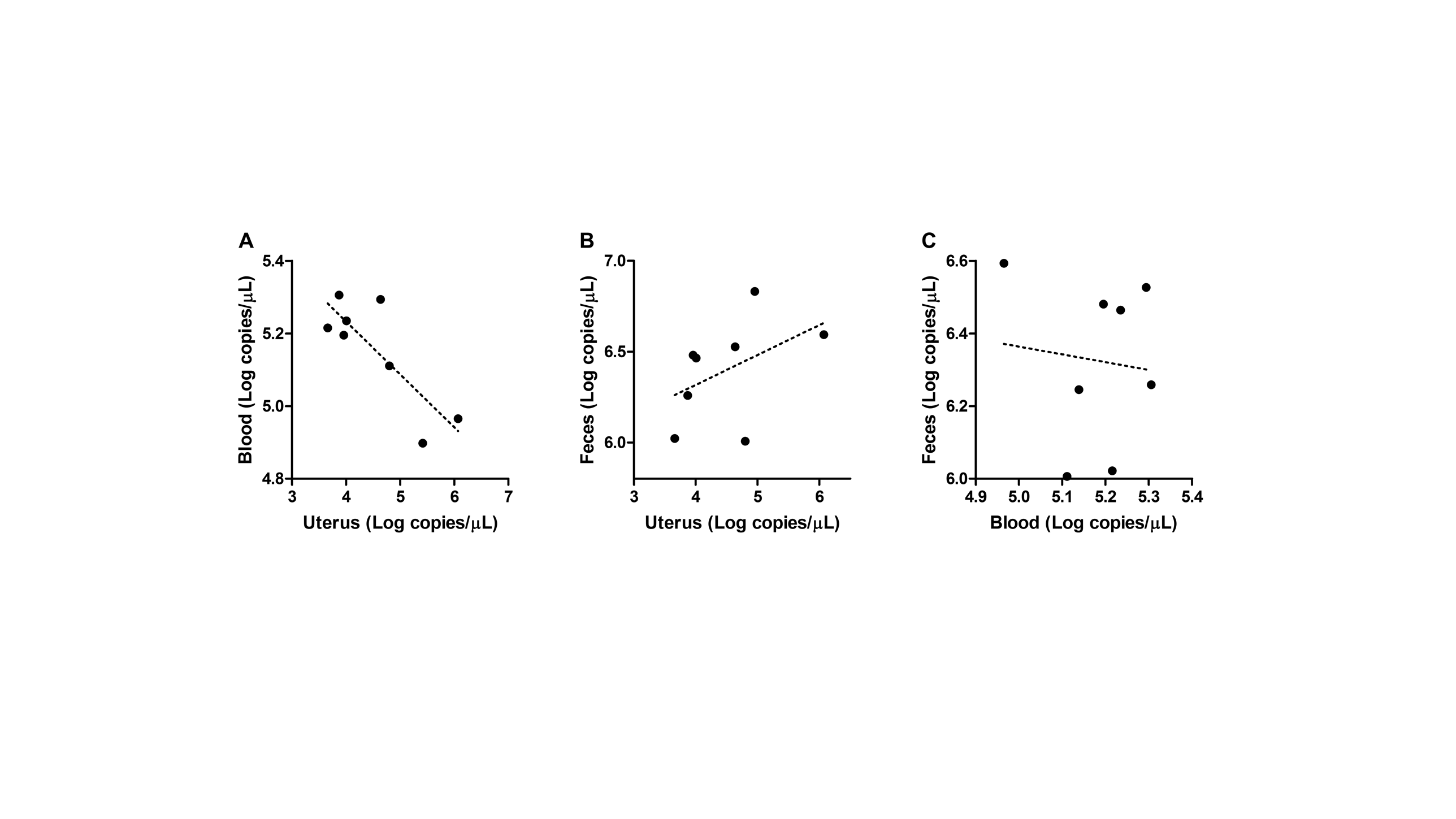

Supplement: Supplementary file 6 — Association between blood and uterine microbiota. (A) Total bacteria in the blood and uterus (Spearman’s r s = 0.69, P = 0.06). (B) Total bacteria in the feces and uterus (Spearman’s r s = 0.60, P = 0.12). (C) Total bacteria in the feces and blood (Spearman’s r s = 0.05, P = 0.91). Blood, feces, and uterine samples were collected from the same individual cows on day 0, and total bacteria were measured using universal primers in ddPCR. (TIFF 1927 kb) [file 40168_2017_328_MOESM6_ESM.tif]

## Slide 1
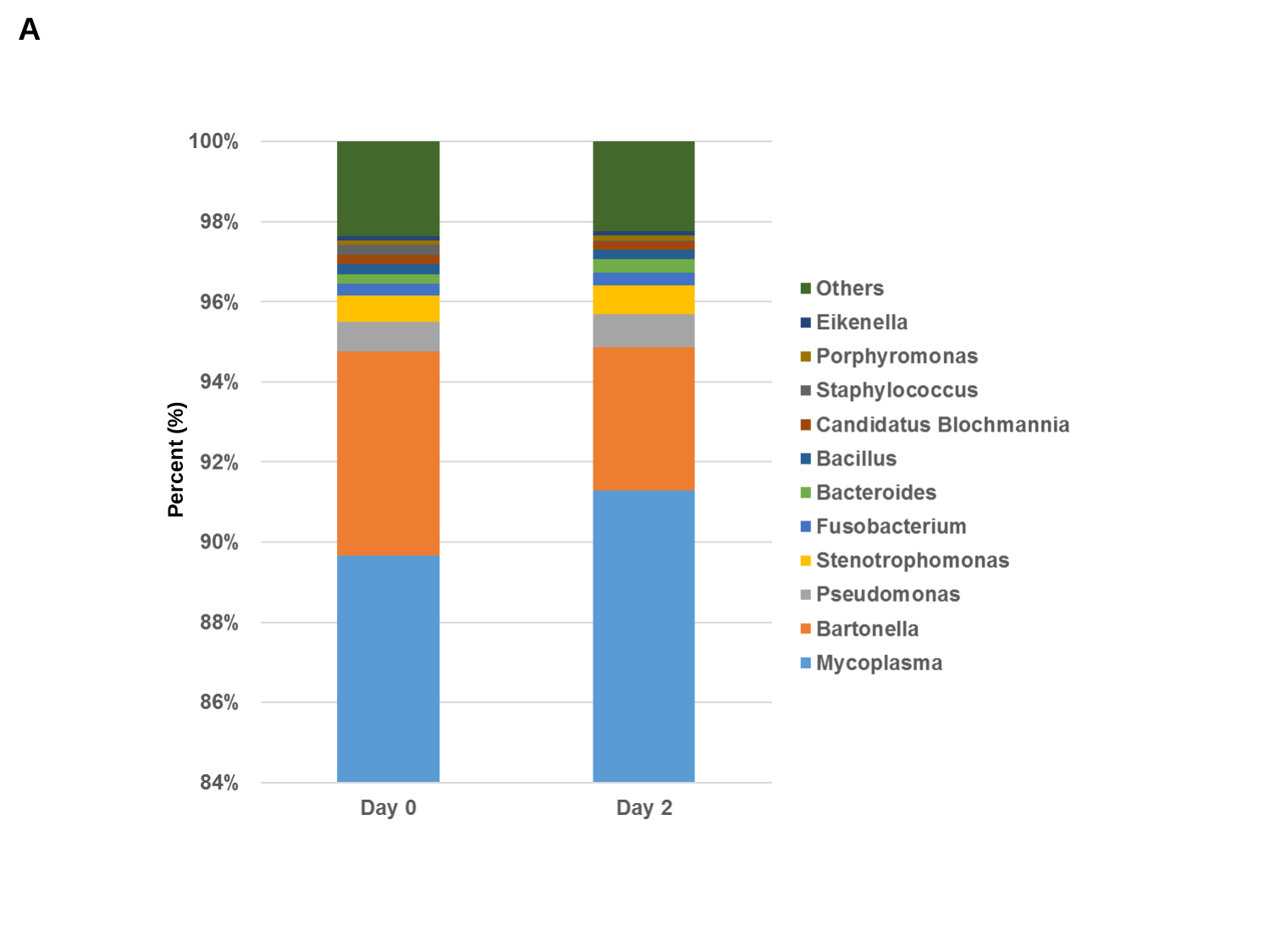

A
Percent (%)

## Slide 2
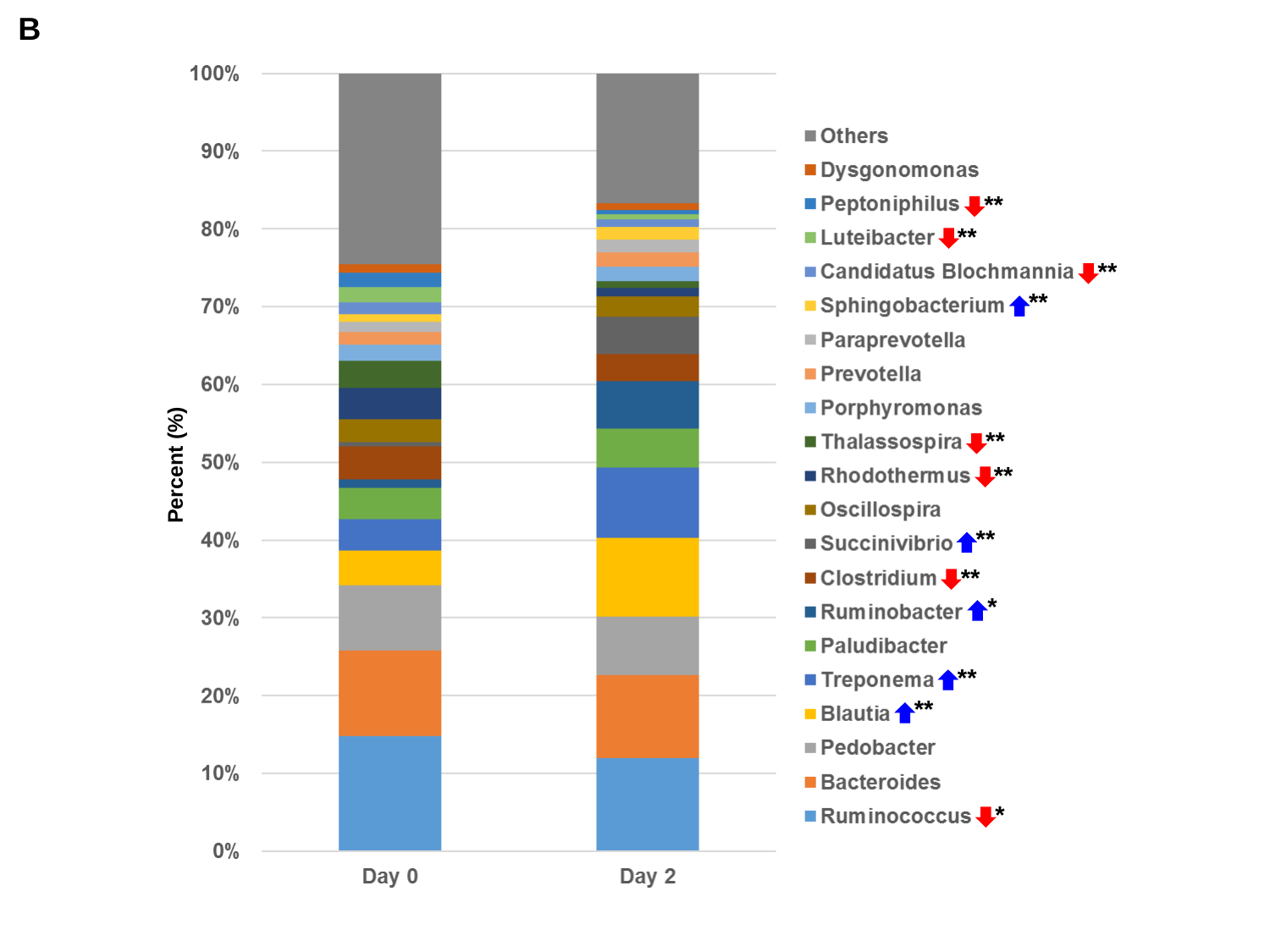

B
**
**
**
**
**
**
**
**
*
**
**
*
Percent (%)

## Slide 3
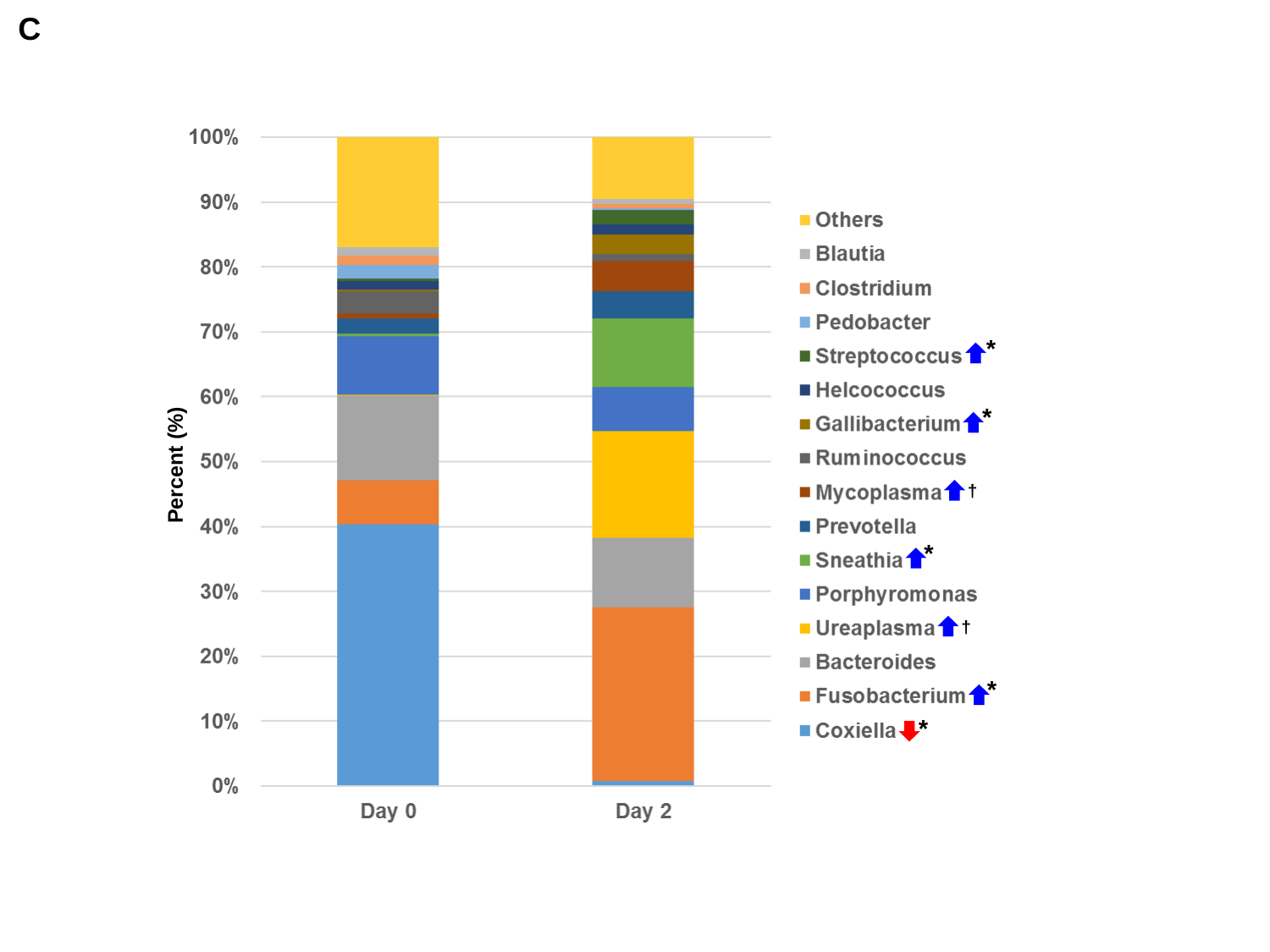

C
*
*
Percent (%)
†
*
†
*
*

Supplement: Supplementary file 7 — Genus-level microbiota abundance. (A) Blood bacterial genera with a relative abundance > 0.1%. (B) Fecal bacterial genera with a relative abundance > 1%. (C) Uterine bacterial genera with a relative abundance > 1%. The asterisks indicate statistical significance between 0 and 2 days postpartum (Wilcoxon test, *P < 0.05, **P < 0.01). The arrows represent increases (blue) and decreases (red) in abundance. (PPTX 147 kb) [file 40168_2017_328_MOESM7_ESM.pptx]
