# Supplementary material for: Blood as a route of transmission of uterine pathogens from the gut to the uterus in cows
Source: Microbiome. 2017 Aug 25;5:109. doi: 10.1186/s40168-017-0328-9 (PMC5574159; doi:10.1186/s40168-017-0328-9)
Supplement: Supplementary file 8 — Primers used in droplet digital PCR. (PDF 145 kb) [file 40168_2017_328_MOESM8_ESM.pdf]

Table S4 Primer information for ddPCR.

Target	Gene	Sequence (5′ - 3′)	Length (bp)	Reference
All bacteria	16S rRNA	F: ACTCCTACGGGAGGCAGCAGT<br>R: TATTACCGCGGCTGCTGGC	180	Clifford et al. (2012) †
Bacteroides heparinolyticus	16S rRNA	F: TAGCTTGCTAAGCCCGATGG<br>R: TACCGGAGTTACCGGAAGGT	72	This study
Fusobacterium necrophorum	*lktA*	F: GATTGGGGGATAGCGGTAAT<br>R: GAGCCTCCACATTTAGTCGC	117	This study

† Clifford RJ, Milillo M, Prestwood J, Quintero R, Zurawski DV, Kwak YI, et al. Detection of bacterial 16S rRNA and identification of four clinically important bacteria by real-time PCR. PLoS One. 2012;7: e48558. doi: 10.1371/journal.pone.0048558 [doi].
